# The Building Blocks for Successful Hub Implementation for Migrant and Refugee Families and Their Children in the First 2000 Days of Life

**DOI:** 10.1111/hex.70082

**Published:** 2025-01-10

**Authors:** Michael Hodgins, Katarina Ostojic, Tania Rimes, Karen Edwards, Kenny Lawson, Mevni Fonseka, Carmen Crespo, Kim Lyle, Ann Dadich, Valsamma Eapen, Rebekah Grace, Melissa Green, Amanda Henry, Nick Hopwood, Catherine Kaplun, Jane Kohlhoff, Shanti Raman, Tracey Szanto, Sue Woolfenden

**Affiliations:** ^1^ Population Child Health Research Group School of Clinical Medicine, UNSW Medicine and Health, University of New South Wales Sydney Australia; ^2^ Community Paediatrics Research Group Faculty of Medicine and Health, The University of Sydney Sydney Australia; ^3^ Child, Youth and Family Services Population and Community Health, South Eastern Sydney Local Health District Sydney Australia; ^4^ Counterpoint Consulting Pty Ltd Sydney Australia; ^5^ Health Economics, Psychiatry and Mental Health University of New South Wales Sydney Australia; ^6^ Translational Health Research Institute Western Sydney University Sydney Australia; ^7^ UNSW Medicine and Health University of New South Wales Sydney Australia; ^8^ Child, Youth and Family Primary and Community Health, North Sydney Local Health District Sydney Australia; ^9^ School of Business, Western Sydney University Sydney Australia; ^10^ Discipline of Psychiatry and Mental Health/School of Clinical Medicine, UNSW Medicine & Health University of New South Wales Sydney Australia; ^11^ Academic Unit of Infant Child and Adolescent Psychiatry Services, South Western Sydney Local Health District Sydney Australia; ^12^ Transforming early Education and Child Health (TeEACH) Western Sydney University Sydney Australia; ^13^ School of Clinical Medicine, Discipline of Psychiatry and Mental Health University of New South Wales Sydney Australia; ^14^ Neuroscience Research Australia Sydney Australia; ^15^ Discipline of Women's Health, School of Clinical Medicine, UNSW Medicine and Health University of New South Wales Sydney Australia; ^16^ University of Technology Sydney Sydney Australia; ^17^ University of Stellenbosch Stellenbosch South Africa; ^18^ Ingham Institute for Applied Medical Research Western Sydney University Sydney Australia; ^19^ Discipline of Psychiatry and Mental Health, School of Clinical Medicine, UNSW Medicine and Health University of New South Wales Sydney Australia; ^20^ Karitane Sydney Australia; ^21^ Community Paediatrics South Western Sydney Local Health District Sydney Australia; ^22^ NSW Agency for Clinical Innovation Sydney Australia; ^23^ Sydney Institute Women, Children and their Families Sydney Australia; ^24^ Community Paediatrics, Sydney Local Health District Sydney Australia

**Keywords:** child and family health, child development, continuity of care, culturally and linguistically diverse, health services, infant health, migrants, resources for child health

## Abstract

**Background and Objective:**

Migrant and refugee women, families, and their children can experience significant language, cultural, and psychosocial barriers to engage with child and family services. Integrated child and family health Hubs are increasingly promoted as a potential solution to address access barriers; however, there is scant literature on how to best implement them with migrant and refugee populations. Our aim was to explore with service providers and consumers the barriers, enablers, and experiences with Hubs and the resulting building blocks required for acceptable Hub implementation for migrant and refugee families.

**Design, Setting and Participants:**

This project was undertaken in Sydney, New South Wales, in communities characterised by cultural diversity. In this qualitative study, we used semi‐structured interviews guided by the consolidated framework for implementation research, with service providers from health and social services (32 participants) and migrant and refugee parents (14 parents) of children who had accessed Hubs.

**Research and Discussion:**

Our initial qualitative data themes were developed into step‐by‐step building blocks, representing a way to address contextual determinants to establish and sustain a Hub that can support migrant and refugee families. These include the setting‐up phase activities of buy‐in and partnership development, which outlines mechanisms to foster collective action and collaboration between health and social services. Following this, our orientation model articulates the need to establish Hub coordination and navigation, activities that enhance a Hub's relevance for migrant and refugee families and ongoing integration mechanisms, such as engagement of same‐language general practitioners. This is the first study to explore the building blocks required for acceptable Hub implementation to meet the needs of migrant and refugee families in the first 2000 days of a child's life—a critical time to optimise child development and health.

**Patient or Public Contribution:**

The research questions were developed based on qualitative research undertaken with Hub participants, community members, and service providers. The original investigator team had a consumer representative who has since relocated and consultation was undertaken with local Hub partner services. The researchers also consulted multicultural health services, including cultural support workers, to ensure research materials were culturally nuanced. Patients or participants have not directly been involved in the current study design.

**Clinical Trial Registration:**

This trial was registered with the Australian New Zealand Clinical Trials (ACTRN12621001088831).

## 1 Introduction

Today, more people than ever live outside their birth country and while many migrate by choice, many move for necessity [1]. Migration has long been considered a key determinant of global health and social development [2, 3, 4]. Of those who have migrated to a new country, parents and their children, particularly those from a marginalised group, have high health needs [5]. Newly immigrated parents bring with them strength and resilience with embedded cultural values, traditional beliefs, and age‐old practices, often influencing their experiences and views in a new social setting [6]. However, without the presence of extended family, parents, particularly mothers, can find themselves isolated and without psychosocial support [6]. Their experiences with accessing healthcare in their country of origin often guide when and how they access care after relocating to a host country [7]. They can experience multiple access barriers including cultural differences, language barriers, poor health literacy, cost of service, and experiences of discrimination [4, 8, 9, 10, 11, 12, 13, 14, 15, 16, 17, 18, 19]. In Australia, children from migrant and refugee families with low English proficiency are 1.5–2 times more likely to commence school without the essential developmental skills needed to flourish [15, 20]. This places them at long‐term risk of inequitable outcomes, including school failure, poor health outcomes, and higher healthcare costs [21, 22, 23, 24, 25, 26, 27, 28]. Early detection and intervention for development, health concerns, and psychosocial support for families by health, early childhood, and social services is imperative to optimise child and family outcomes.

Integrated child and family health (CFH) Hubs are increasingly promoted as a mechanism to achieve reduced access barriers to health and social services to improve child and family outcomes [29, 30]. The term ‘Hub’ here refers to a central location or entity that coordinates various aspects of healthcare delivery or services. This term is likely derived from its common usage in transportation and technology, where a Hub serves as a central point of connection or activity. The term has been approved by migrant and refugee families as part of previous consultation work led by the authors of this paper [31]. Hubs involve physically co‐locating or virtually connecting health and social services with supported care navigation and shared referral pathways [32, 33]. Within these Hubs, integration between services is prioritised so that people receive the care they need, when they need it, in ways that are user‐friendly, achieve the desired results, and provide value for money [34]. Hubs are more than a form of interdisciplinary care with collaboration between clinicians from different disciplines. The integrated care that takes place in a Hub takes a broader approach, emphasising coordination and integration of services across the entire healthcare, social, and early childhood systems to provide seamless care to children and their families [29, 30]. Hubs can increase service engagement offering greater opportunities to identify and address families' developmental concerns and psychosocial needs [35, 36]. While our understanding of how Hubs should operate has progressed using theoretical frameworks such as collective impact [37, 38], the operational components required for successful Hub implementation that support the unique needs of migrant and refugee families have not been explored with the existing frameworks [39, 40]. Thus, the aim of this study was to explore with service providers (SPs) and consumers the barriers, enablers, and experiences with Hubs and the resulting building blocks required for acceptable successful Hub implementation for migrant and refugee families. This will inform the future development of a Hub implementation toolkit to translate Hub principles into concrete actions.

## 2 Design

### 2.1 Ethics

Ethical approval was granted by the South Eastern Sydney Local Health District Human Research Ethics Committee in July 2021 (Project ID: 2020/ETH03295).

### 2.2 Theoretical Framework

The theoretical framework used in this study was the Consolidated Framework for Implementation Research (CFIR) [41]. The CFIR offers ‘an overarching typology to promote implementation theory development and verification about what works where and why across multiple contexts’ [41]. The CFIR identifies five major domains (innovation, outer setting, inner setting, individuals, and implementation process) to guide the consideration and assessment of factors that can impact intervention implementation and effectiveness.

### 2.3 Setting and Participants

This project was undertaken in Sydney, New South Wales, with three local health district sites—South Eastern Sydney, South Western Sydney, and Northern Sydney. In these areas, between 38% and 49% of residents speak a language other than English at home [42]. The sites were selected as they participated in a larger study evaluating the impact, implementation, and cost–benefit of Hubs for migrant and refugee women and their infants [43]. The Hub at each site included CFH services, specifically a CFH nursing service, as well as other health and social services. SP participants (*n* = 32) included representatives of CFH services (20), social SPs (8), and local health district managers (4). This sample comprised CFH nurses, nurse unit managers, health service directors, social service directors, clinical nurse consultants, a Hub coordinator, and a Hub navigator. Consumer participants included 14 migrant and refugee parents (12 mothers and two fathers) who had attended the Hubs in South Eastern and South Western Sydney. The consumer participants' countries of birth included Bangladesh, Nepal, Mongolia, Vietnam, and Iraq.

### 2.4 Data Collection and Analysis

Data collection and analysis focused on exploring experiences with successful Hub delivery for migrant and refugee women and their children to conceptualise the building blocks and involved three iterative phases. During Phase 1, SPs were purposively recruited from the three local health districts based on their prospective participation in a larger pragmatic trial of Hubs for migrant and refugee women and their children [43]. Participants participated in semi‐structured interviews, conducted by three authors (M.H., M.F., and K.E.). Two had extensive qualitative research experience (M.H. and K.E.), and one was an honours student, supervised to conduct interviews (M.F.). Guided by the CFIR, the semi‐structured interviews investigated the barriers and facilitators to implement Hubs for migrant and refugee families (see Appendices [Link], [Link], [Link]). All interviews were conducted online via Microsoft Teams or Zoom. Interviews were recorded and transcribed verbatim. Interview data were thematically analysed [44]. Three researchers conducted the analysis (M.H., M.F., and C.C.), which involved familiarisation with the data set, generating initial codes via line‐by‐line coding, and collating data relevant to each code. An initial coding framework was developed by one author (M.F.), which was refined through discussion with two other authors (M.H. and C.C.) and further refined with input from other investigators (S.W., T.R., A.H., and K.O.).

Phase 2 involved presenting this preliminary analysis, which outlined the barriers and enablers to implementing Hubs, via workshops for both the co‐investigators of the study and the sites. The workshops served to conceptualise the core components for Hub implementation for migrant and refugee families to guide the implementation evaluation of Hubs in the larger pragmatic trial [43]. Six workshops were facilitated, two per participating site and two with the investigators. Workshops were recorded and minutes were created based on the discussion. Based on interview and workshop data, two authors (M.H. and K.E.), with input from the broader team, developed five core components—or building blocks—to implement Hubs for migrant and refugee families. These building blocks were developed by applying a processual lens to the analysis, organising themes according to a temporal pattern of Hub development. These themes are summarised as the step‐by‐step building blocks required to define, develop, and sustain a successful Hub according to our data set.

In Phase 3, the SP analysis was supplemented by interviews with migrant and refugee parents who had attended Hubs as part of the larger pragmatic trial [43]. Interviews explored parent experiences with and preferences for the Hub model. Twelve mothers and two male partners were purposefully recruited from the trial participants who had attended the Hubs. Parents participated in an interview (between 10 and 30 min) either face to face or via telephone with a research assistant, supported by the first author (M.H.). Interviews were conducted in English with an interpreter service offered (none of the participants accepted the use of an interpreter). Supplementary analysis of this data focused on exploring the experiences of migrant and refugee parents attending Hubs. This analysis was incorporated with the provider perspectives to determine the compatibility of perspectives between providers and consumers of Hubs in the conceptualisation of the building blocks for Hub implementation.

## 3 Results

**Figure 1:**
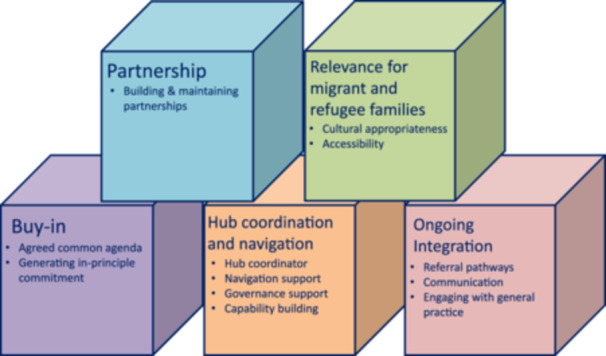
Building blocks to develop and sustain child and family Hubs.

In this section, we detail key themes related to the building blocks (Figure 1).

### 3.1 Buy‐In

According to SPs, one of the keys to Hub development is bolstering Hub stakeholder support, namely, the partner services involved in the Hub. SPs commonly discussed the challenges of negotiating an unfamiliar health system in a new country for migrant and refugee families. They noted that families commonly grappled with financial barriers when accessing services, exacerbated by ineligibility for free or subsidised healthcare via a national insurance scheme.

I think many families have complex psychosocial challenges as well and I think basically having the right services there at the right time for the families when they need them is essential.

(Health service manager)

With different countries of origin, each community had specific needs that could not necessarily be addressed by a single approach. Creating the conditions for this model of service provision requires buy‐in from a composition of appropriate services (including health, social services, education, and early childcare) within a Hub, all of which are committed to the needs of migrant and refugee families.

#### 3.1.1 A Common Agenda

Achieving stakeholder agreement on a Hub's agenda was described as the necessary first step to determine the viability of a Hub. This requires a collaborative and consistent understanding across different SPs of the role of each service in delivering a Hub's agenda:

It's about getting agreement on what things you can do … and what things you might try and [how to] work together with certain client groups.

(Health service manager)

Conflicting aims between different services, such as health, early childhood, and social services, can hinder collaboration; this might be due to different service goals and relatedly different levels of commitment towards Hubs:

Because, at the end of the day, we're all wanting the same, we're wanting better outcomes. And all of us have a different role. So, no duplication.

(Social service manager)

#### 3.1.2 Generating In‐Principle Commitment

Beyond agreeing on the Hub goal, SPs noted the need to establish commitment amongst Hub partners. At a minimum, this should involve a commitment to a set of common principles and/or ways of working to better meet the needs of families or more formally as memoranda of understanding. Engaging leaders from the partner services using working parties enabled providers to build persistent and open communication channels between services. Establishing clear goals with the partnering services regarding engagement and role expectations was important for Hub impact and sustainability:

Just being really open and working on those collaborative partnerships and really not trying to control other services and just being really aware of that and letting everyone have their input.

(Healthcare staff)

### 3.2 Partnership

#### 3.2.1 Building and Maintaining Partnerships

Strengthening partnerships between health and social SPs and consumers is an important, albeit challenging, step when creating a Hub for migrant and refugee families. Effective partnership required clear leadership and worker commitment to the Hub concept:

I think, for it to really work for the client, there needs to be greater transparency and collaboration and generosity, with how services really work together.

(Social service manager)

Trusting relationships amongst partners are formed over time. Staffing changes can result in sudden disengagement of partner services, which can compromise partnerships, and clear service leadership is required to address this:

Agencies that have left due to a change in worker … actually had impacts for us at … the Hub because they are on our promotional material. So, we really need a manager at some level to sort of say… ‘We are committed to this’.

(Health service manager)

### 3.3 Hub Coordination and Navigation

According to SP participants, it is critical to keep families engaged within a system that understands their needs. SPs outlined the importance of routine contact with families and ‘clear communication pathways between services'. Central to meeting this need involved employing a Hub coordinator to manage the Hub, employing a Hub navigator, having clear governance arrangements, and building workforce capability to work in an integrated way.

#### 3.3.1 Hub Coordinator

Many participants noted that a dedicated Hub coordinator was essential to facilitate and coordinate Hub development and continue to support sustainability. A Hub coordinator was described by the SPs as the driving force behind a Hub ‘keep[ing] the ball rolling]’ and managing relationships. One health manager described a coordinator as:

You really need someone who is in tune with all the subtleties that often are completely missed. I think without that everyone is busy and everyone gets back into their own organisations. I think without someone who brings you together all the time it's hard to keep the momentum going.

(Health service manager)

Participants noted the time and work required, particularly during establishment, to coordinate between services and facilitate foundational work, which could not be added to the work of an existing staff member. A Hub coordinator differs from a care navigator, operating at a more senior level with decision‐making delegations. A Hub coordinator's tasks might involve facilitating meetings between partners, setting up governance structures, managing facility issues, developing referral pathways, securing funding, and connecting with other potential partners. This work required a ‘skilled communicator who can continuously be working with all the different agencies to talk about things before they become issues’ as one health service manager suggested.

#### 3.3.2 Navigation Support

Several SPs considered a Hub navigator role essential for engaging families and supporting their access and transition between Hub services. Participants highlighted the importance of a Hub navigator to support increased trust and rapport with migrant and refugee families.

I think a key person who is supporting that navigation is essential, who is supporting that connection to other services and also doing that soft referral. Someone who has a rapport with the families … if there's trust in the navigator there will be trust in the services that they are referred to.

(Health service manager)

SPs and parents also identified that close relationships between the maternity and CFH services at the Hub led to the earlier and more efficient identification of potentially vulnerable families and better linkage of these families.

Families pretty much know my face. They know my name and they know who I am, whether it's through programs or through other services, they are quite familiar with whom to seek support from or who might be able to give them some guidance as to what their next move is.

(Hub navigator)

#### 3.3.3 Governance Support

The senior governance group formed from Hub partners should lead the development and ongoing work, collaboratively developing solutions to issues, sourcing resources, and maintaining senior‐level commitment to the Hub:

Setting up good governance is a key principle of working, which, at times, we have to come back to when we lose our way, which is quite often. We have to [come back to] why we are here and what we are doing.

(Health service manager)

#### 3.3.4 Capability Building

Participants noted that Hub staff members’ professional development skills should be addressed if a Hub was to be successfully implemented and sustained:

[Working in a Hub model] is nothing like anything I've probably ever done before … there's often quite a bit of flexibility around [workload].

(Child and family health nurse)

Participants noted areas of consideration, including flexible management to support different ways of working, tools for interagency collaboration, shared protocols and processes to support integration, trauma‐informed care and clinical supervision:

It's so important having a management team that's willing to look at other ways of working.

(Health service manager)

Hub parents held Hub staff members in high regard for their friendliness and inclusiveness, putting parents at ease while attending appointments. Parents cited care and concern for the family, thoughtfulness while scheduling appointments, and patience when the mothers pose questions and doubts as valuable:

[The nurse] emails, it's easy. We don't have to go out and reach out for help… Everything seems smooth.

(Parent)

### 3.4 Relevance for Migrant and Refugee Families

#### 3.4.1 Cultural Appropriateness

Culturally responsive care was seen as a crucial factor in overcoming the unmet needs in this community and ensuring sustained engagement with the Hub. Key barriers to culturally safe care were the challenges in providing in‐language support services and the limitations of a one‐size‐fits‐all approach. While the bilingual parents did not explicitly refer to the benefit of interpreters, their expressed preference for in‐language general practitioner (GP) consultations suggests that interpreter services or bilingual SPs should be a priority for emerging Hubs.

The language speaking stuff is very important. [Some migrant groups] have language needs that are very complex. And they would prefer to go to someone who speaks their language.

(Health service manager)

In terms of culturally safe and welcoming care, participants identified the importance of the physical space. This included the external appearance of a Hub, noting how often corporate aesthetics can be unwelcoming. Making the physical space culturally safe and welcoming, engaging in patient and public involvement, and conducting outreach where necessary can aid relevance:

[We are providing] an integrated, almost one‐stop‐shop [service] in a way that is culturally safe and responsive to their needs and located in a geographic spot that is accessible for them.

(Health service manager)

#### 3.4.2 Accessibility

Creating accessible and engaging services by creating consistent touchpoints for families, with a trusting and trauma‐informed relationship built over time, was crucial to addressing the challenges of migrant and refugee families.

In addition to the health services provided by the CFH nurse, the Hubs offered the family support and development officer, community paediatrician, playgroups, preparation for preschool, community dental services, breastfeeding support, mental healthcare, speech therapy, disability services, and occupational therapy. Parents appreciated this:

It's a good experience because we get to know about our baby's health, how he's doing, about his height, weight and anything related to health. And the nurse there is so helpful. And she makes the baby comfortable first and takes the weight and other stuff. And they also ask about our feelings, how we are feeling, how we are doing. It's a great experience.

(Parent)

According to the SPs, ‘being available’ is key to sustaining engagement with migrant and refugee families. This might involve creating flexible ways to engage with services such as soft entry points, being responsive and proactive, being ‘friendly’, and ‘go[ing] out into the community to seek families’. Parents were also pleased when care included both parents. Parents clearly described that their affinity towards the Hub staff, particularly the CFH nurse, was a key positive influence in their ongoing attendance of Hub appointments:

Give me the text message like, ‘Oh, I have an appointment this time’ and then, if I can come or not come if I'm busy that day, or my kid is not well that day, I could just text her back and she replies straight away, which is a good thing.

(Parent)

The migrant and refugee parents consistently stated that the proximity of the Hubs from their homes affected their decision to attend the Hubs:

It's nice to have this five‐minute drive, it's quite convenient.

(Parent)

SPs also emphasised the importance of establishing trusting relationships with migrant and refugee communities. They noted the importance of ‘word of mouth and the trust of the community’ for engagement. Home visits in the early postnatal period and antenatal engagement with migrant and refugee parents were also opportunistic methods to understand the home dynamics and the specific needs of the families, further supporting family‐centred care. SPs proposed that engagement would be improved with greater parental health literacy, particularly about services that promoted early child development. They indicated that limited knowledge within the community increased distrust of health and social services. Trust was facilitated through community and consumer engagement and strategies were developed to incorporate this on an ongoing basis:

They are the cohort that are hard to engage, and you know they've come from a lot of countries where this service was not there. So, they don't understand the service and they are not very trusting of the service, they may have had some sort of trauma growing up with health service.

(Health service manager)

### 3.5 Ongoing Integration

To sustain a Hub and the pathways and partnerships involved creating and maintaining a centralised referral pathway document, regular partner meetings, methods for sharing client information, and engagement with general practice.

#### 3.5.1 Referral Pathways

SPs noted that Hub models were inherently complex, involving vertical and horizontal integration across sectors. A central point of Hubs is to support cross‐referral between partner services. It is vital that Hubs identify the relevant services within the Hub, the intake criteria for the services, and the formal mechanisms for referring clients to these services:

Knowing who the partners are and knowing whom your stakeholders are, your partners and knowing what their capacity is at accepting and making referrals. What can they offer to each other?

(Healthcare staff)

Understanding the referral methods, which include warm handovers and soft referrals, facilitated better access to different services. Warm handovers, which involve a service navigator guiding the family to better access to care, according to the recognised needs, were acknowledged to be a great facilitator for the referral process:

To ideally work closely with the social SPs to support the families, looking at referring back and forth… keeping those networks and the referrals is really useful for the model to survive because it's not just about one service. They've all got their own roles to play.

(Health service manager)

This was also supported by soft referrals, which mainly involved a non‐health service, such as a playgroup, referring to the CFH nurses within Hubs for specific needs.

#### 3.5.2 Communication

Hub meetings are held as part of the Hub daily operations, including huddles between SPs, to discuss the Hub operation and clients with complex needs. Formal mechanisms, like Hub meetings, form the backbone of communication between partners and require consistent representation from partner services to ensure transparency and connectivity:

We have a monthly team meeting, and we talk about vulnerable families that we feel we can help out for everyone in the Hub and we get the input from the other healthcare professionals as well, and how everyone can support as a team. That's the way we can be on the same page for the family.

(Healthcare staff)

Many SPs agreed that access to shared client information supports a more integrated model of care; however, many also noted the challenges in achieving integrated client records.

#### 3.5.3 Engaging With General Practice

SPs indicated that engaging general practices with a Hub was challenging. Most SPs also identified that migrant and refugee communities preferred GP care due to strong existing relationships with GPs who spoke the language of their country of origin and familiarity with the role of GPs as opposed to CFH services.

They only know their GPs and of course the GP speaks the language so it's easier to access… The GP also does the immunisations which we don't.

(Health service manager)

As stated, parents perceived CFH services as novel and difficult to differentiate from the health services provided by the GP. SP participants noted the ‘disconnect’ or lack of understanding about the difference between GP and CFH services, also noting their preference for GPs. Parents supported this, stating they often prioritised GP visits over CFH nurses, especially if they had more than one child and wanted to avoid appointment duplication. Parents reported that they trusted the GPs for ‘medical’ health issues more than the CFH nurses. One mother expressed the convenience of attending their GP for developmental checks, vaccinations, and health concerns in one:

We went to GP check‐up for six months and vaccination. And we've been sick lately. So, we just call GP and check up on him … when you go to the GP you have the vaccination and to do all the checks all over again.

(Parent)

## 4 Discussion

This is the first study to explore the barriers, enablers, and experiences of Hub for SPs and parents and conceptualise the building blocks for successful Hub implementation to meet the unique needs of migrant and refugee families. Previous studies have described cultural, linguistic, and financial barriers to accessing services and the feasibility and acceptability of outreach CFH services into multicultural playgroups [15, 35]. However, none—to our knowledge—have conceptualised the key components for successful Hub implementation specifically for migrant and refugee children and their families using this data or the CFIR.

Our findings support the emerging literature on the importance of fully integrated care, rather than mere co‐location, with shared systems focusing on collaborative interdisciplinary work and clarity of service roles [45, 46]. To move from co‐location to integration requires clear and open communication between services, a service coordinator to facilitate collaboration, and openness from services to help families navigate and understand the responsibilities and limitations of each service [47, 48, 49, 50].

We found that proactive service engagement was viewed positively, by parents and SPs, particularly because many migrant and refugee parents were away from their families or core support system that would usually guide them while child rearing. CFH services are often novel to migrant and refugee families, who do not have comparable services in their home countries. It is key therefore that these services take time to build trust and rapport with families, provide in‐language support services, ensure a warm and friendly waiting room, have sensitive navigation support, and practice trauma‐informed care. With different countries of origin, each community had specific needs that cannot be addressed by a single approach. Thus, creating a trauma‐informed Hub requires careful thought and appropriate buy‐in from a composition of services (including health, social services, early childhood) within a Hub, all of which are committed to meeting the needs of migrant and refugee families. This supports existing literature that in‐language support services, including cross‐cultural workers, Hub‐based interpreters, and staff members who are bilingual, cultural and in‐language support, are all key to trauma‐informed ways of working with migrant and refugee families [51, 52].

Our research has highlighted that GP engagement is a clear priority for ongoing integration. The inability to offer childhood vaccinations hindered family engagement with CFH nurses, increasing a preference for GP services. Creating and sustaining change in primary care is challenging, given they are an ‘underappreciated and underfunded specialty’ relying on federal funding in Australia for the individual practitioner rather than supporting working in integrated models [53]. There is emerging evidence of the efficacy and acceptability of integrated paediatric and primary care involving co‐location of GPs and child health specialists within general practices [54, 55].

Recent formative Hub work in Australia to address childhood adversity has highlighted the importance of navigation support, workforce development, clear referral pathways, and partnerships [56]. Our findings add that clear structures for Hub governance and partnerships which are overseen by a Hub coordinator with authority and decision‐making delegations are essential for Hub implementation. Capability building of the existing CFH nurse workforce is also key to workforce development. Hubs require a different way of working, representing a challenge for staff members who are accustomed to providing standalone services. Building this capability requires clear organisational commitment to culture change driven by the Hub governance model. This includes a commitment to address the organisational barriers of limited and piecemeal funding, the difficulty of recruiting and retaining a skilled workforce, and siloed data with continuous evaluation and quality improvement initiatives [56, 57].

### 4.1 Implications for Service Delivery and Research

Based on our findings, there are key actions that are required to enable the Hub building blocks. To institutionalise support and partnership, Hub partners should develop a memorandum of understanding or similar, detailing partner commitments, including representation at meetings, service commitment to activities, and training. Additionally, senior‐level delegations are needed to provide an authorising environment for the resources required to establish and sustain a Hub. This senior‐level governance supports middle and frontline managers in their day‐to‐day governance roles. To enable integration requires a programme of workforce development to support integrated models of care and the often psychologically demanding nature of working in these models [30]. These and other developmental activities should be included in a staff development plan for the Hub. We would also argue that, without clear and sustainable funding for a Hub coordinator, a Hub will not thrive as Hub coordinators are the lynchpin in ensuring Hub success. A key challenge for all Hubs working with migrant and refugee communities is engagement with and funding models that support partnership with GPs who can provide in‐language medical support, medical care, and vaccinations to Hub attendees.

## 5 Conclusion

In this article, we described the building blocks needed for Hub implementation to support migrant and refugee families from the perspective of SPs and parents. By building and sustaining Hubs delivered equitably, we can improve growth and developmental outcomes in the first 2000 days, resulting in substantial flow‐on effects for longer term educational, social, and health outcomes.

## Author Contributions

M.H., S.W., T.R., K.O., K.L., A.D., V.E. and A.H. conceptualised and designed the study. MV, MH and KE collected data and undertook the analysis. M.H., M.V. and K.E. developed the initial draft of the paper, which was refined by T.R., S.W., K.O., K.L., A.D., V.E., A.H., C.C., R.G., M.G., N.H., C.K., J.K., S.R. and T.S. All authors reviewed the final manuscript on behalf of the FDCC Collaborative Group.

## Ethics Statement

Ethical approval was granted by the South Eastern Sydney Local Health District (SESLHD) (2020/ETH03295).

## Conflicts of Interest

The authors declare no conflicts of interest.

## Supporting information

Supporting information.

Supporting information.

Supporting information.

## Data Availability

Data are available on contacting the authors.
