# Supplementary material for: The Building Blocks for Successful Hub Implementation for Migrant and Refugee Families and Their Children in the First 2000 Days of Life
Source: Health Expect. 2025 Jan 10;28(1):e70082. doi: 10.1111/hex.70082 (PMC11721473; doi:10.1111/hex.70082)
Supplement: Supplementary file 1 — Supporting information. [file HEX-28-e70082-s002.docx]

**Appendix 1 – Hub Manager Interview Guide**

***Introductions***

***Confirm consent and re-iterate that the interview will be recorded; transcripts will be de-identified and any quotes used will not be attributed by name***

**General**

What do you believe were the incentives or motivations for implementing child and family health Hubs?

**Implementation Process**

What is your impression about how practical it was/is to implement the Hub?

What kinds of infrastructure changes had/have to be made to get the Hubs running effectively? *Prompts: changing location, extra staff, new technology.*

Do LHDs have the resources to run Hubs effectively? What extra resources do you think they need?

What do you believe could have been done differently to improve the implementation of the FDCC Hubs?

**Fidelity and unexpected consequences**

What have been the consequences (positive or negative) of the FDCC Hubs? *Prompt: Were there any surprising effects on practice or outcomes that you are aware of?*

Does the Hub address the needs of Migrant and refugee families? *Prompts: Is it in the right location? Does it have the right kinds of services? How do you deliver culturally sensitive practice?*

Do you believe that the FDCC Hub model has had financial implications for migrant and refugee child and family health?

*If yes*, tell us about these financial implications?

**Effects of collective impact on knowledge, confidence and practice**

What are you perceptions of the collaboration between NGO/Health staff? *Prompts: Communication? Sharing of resources?*

To what extent do you feel the Hubs support the needs of migrant and refugee families?

**Sustainability**

When the trial is finished, do you believe that a Hub or similar model will continue? *Please provide reasons*.

What incentives do you think are needed to continue the Hub model?

What is your opinion about implementing a Child and Family Health Hub in other contexts, e.g. in a rural or regional area?
