# Supplementary material for: The Building Blocks for Successful Hub Implementation for Migrant and Refugee Families and Their Children in the First 2000 Days of Life
Source: Health Expect. 2025 Jan 10;28(1):e70082. doi: 10.1111/hex.70082 (PMC11721473; doi:10.1111/hex.70082)
Supplement: Supplementary file 2 — Supporting information. [file HEX-28-e70082-s001.docx]

**Appendix 2 – Hub Staff Interview Guide**

***Introductions***

***Confirm consent and re-iterate that the interview will be recorded; transcripts will be de-identified and any quotes used will not be attributed by name***

**General**

What do you believe were the incentives or motivations for implementing the Hub in your LHD?

**Implementation Process**

What is your impression about how practical it was/is to implement the Hub?

What kinds of infrastructure changes had to be made to get the Hubs running effectively? *Prompts: changing location, extra staff, new technology.*

Do you have the resources to run Hubs effectively? What extra resources are needed?

Does the Hub model suit how you would like to practice? If so, how? If not, why not? *Prompt/alt: How and to what extent did/does the FDCC Hub impact on the usual way your practice works?*

What do you believe could have been done differently to improve the implementation of the FDCC Hub?

Are your managers supportive of the Hub model? How do you know?

**Fidelity and unexpected consequences**

Before working within a Hub model what were your expectations?

What have been the consequences (positive or negative) of working within a hub model? *Prompt: Were there any surprising effects on your practice?*

Does the Hub address the needs of Migrant and refugee families? *Prompts: Is it in the right location? Does it have the right kinds of services? How do you deliver culturally sensitive practice?*

Do you believe that the FDCC Hub model has had financial implications for your practice?

*If yes*, tell us about these financial implications?

**Effects of collective impact on knowledge, confidence and practice**

How often did/do you liaise with other kinds of service providers? *I.e. NGO staff liaising with CFH staff or vice versa.*

While working in the Hubs what has it been like to work alongside NGO/Health staff? *Prompts: How do you communicate? Do you share resources?*

How well do local GPs and practices support migrant and refugee child and family health?

To what extent do you feel you are better able to support the needs of migrant and refugee families because of the Hub model?

**Changes in attitudes**

Has working with a Hub model changed your attitudes to providing care to migrant and refugee families? If so in what ways?

Do you think the attitudes of colleagues in your practice have changed? *If so* in what ways?

**Sustainability**

When the trial is finished, do you believe that your practice will continue working within a Hub or similar model? *Please provide reasons*.

What incentives do you think are needed to continue the Hub model for your practice?

What is your opinion about implementing a Child and Family Health Hub in contexts different to yours, e.g. in a rural or regional area?
