# Supplementary material for: The Building Blocks for Successful Hub Implementation for Migrant and Refugee Families and Their Children in the First 2000 Days of Life
Source: Health Expect. 2025 Jan 10;28(1):e70082. doi: 10.1111/hex.70082 (PMC11721473; doi:10.1111/hex.70082)
Supplement: Supplementary file 3 — Supporting information. [file HEX-28-e70082-s003.docx]

**Appendix 3 Parent Interview Guide**

1. Describe your experience of attending the [insert site name- South East Sydney/ South West Sydney ] Hub. *Prompt who have you been seeing here?*
2. What activities/services have you accessed since attending the Hub? *Prompts: Tell me about your experience with these services? Did they attend to your or your child’s needs? What services within the Hub are you aware of?*  *Has the CFHN connected you with services?*
3. Are you attending any other child or family health services (GP, paediatrician, allied health, etc?)
4. Is the Hub convenient for you? Why/why not?
5. What do you like/dislike about the Hub?

*Prompts: Location, available services, culturally appropriate?*

1. What would you like to see done better next time you attend the Hub?
2. Do you think you’ll continue to come to the Hub in the long-term?
3. Did participating in the study encourage you to attend the Hub? What child health services would you have accessed if you weren’t participating in the study?
